# Anomalous Dynamics in Macromolecular Liquids

**DOI:** 10.3390/polym14050856

**Published:** 2022-02-22

**Authors:** Marina G. Guenza

**Affiliations:** Department of Chemistry and Biochemistry, Institute for Fundamental Science and Material Science Institute, University of Oregon, Eugene, OR 97403, USA; mguenza@uoregon.edu

**Keywords:** anomalous subdiffusive dynamics, unentangled polymers, Rouse equation, cooperative many-chain dynamics

## Abstract

Macromolecular liquids display short-time anomalous behaviors in disagreement with conventional single-molecule mean-field theories. In this study, we analyze the behavior of the simplest but most realistic macromolecular system that displays anomalous dynamics, i.e., a melt of short homopolymer chains, starting from molecular dynamics simulation trajectories. Our study sheds some light on the microscopic molecular mechanisms responsible for the observed anomalous behavior. The relevance of the correlation hole, a unique property of polymer liquids, in relation to the observed subdiffusive dynamics, naturally emerges from the analysis of the van Hove distribution functions and other properties.

## 1. Introduction

The dynamics of synthetic and natural macromolecular fluids (e.g., polymer melts [1], proteins [2,3,4,5], DNAs [6,7,8], and cellular microfilaments [9]) is described conventionally by mean-field theories of single-molecule motion. The underlying assumption in these approaches is that the relaxation of the surrounding fluid occurs on a different timescale compared to the dynamics of the tagged molecule. When this hypothesis holds, it is possible to derive a single-chain equation of motion by projecting, through Mori–Zwanzig techniques [10], the dynamics of the entire fluid onto a set of slow relevant variables (here, the coordinates of the tagged chain). The single-chain equation of motion is a generalized Langevin equation that, in the limit of completely flexible polymers and when the memory function is discarded, reduces to the popular Rouse equation of motion for the dynamics of unentangled polymer melts [11,12,13]. The Rouse model provides a simple description of chain dynamics for long polymer chains (the degree of polymerization is assumed to go to infinity), while predicting the scaling exponents of chain dynamics in remarkable agreement with experiments [1,14]. To introduce a more realistic description of the polymer chains, the Rouse model has been modified by adopting intramolecular chain distributions that include local semiflexibility [15], which accounts for the complex nature of local energy barriers [16,17], and monomer-dependent friction coefficients [18,19]. Furthermore, monomer fluctuations that are harmonic in a body-centered description of the dynamics, are intrinsically anharmonic in the lab reference system of a Rouse-like description due to the coupling of internal fluctuations with the molecular rotational and translational dynamic [20,21]. Summarizing, the simple mean-field formalism of the Rouse model provides a useful general description of the polymer dynamics, which one can improve by including a more realistic molecular description than the typical chain of beads connected by harmonic springs. Nevertheless, the fundamental hypothesis of the separation of timescales that motivates the Rouse formalism, i.e., a Langevin equation in the lab-frame for the monomer (beads) coordinates, becomes questionable when describing systems where the “solute” and the “solvent” relax on the same timescale [22].

The hypothesis of the separation of timescales for the dynamics of one polymer chain with respect to its surroundings, which is the fundamental hypothesis in the Mori–Zwanzig projection operator method, holds for macromolecular systems in dilute solutions of small-molecule solvents, where the slowest timescale of relaxation is reduced by the small size of the solvent molecules. However, its validity may require further scrutiny for concentrated polymer solutions [22], melts [14,23,24], blends [25,26], and particles in crowded cellular environments [27,28]. For these systems, experimental and computational data show discrepancies with the theoretical predictions, which are conventionally termed “anomalous dynamics”. In this work, we investigate the microscopic physical picture behind anomalous dynamics and relate this picture to a theoretical model, i.e., an extended Rouse equation, for a subensemble of slowly moving, interacting chains undergoing cooperative dynamics, where “solute” and “solvent” relax on the same timescale [29,30,31]. We focus on the simplest realistic macromolecular fluid that exhibits this general behavior, i.e., a melt of short homopolymer chains well above its glass transition. From the analysis of molecular dynamics (MD) simulations of unentangled polyethylene (PE) melts, we propose an intuitive physical explanation for the observed anomalous dynamics.

## 2. Materials and Methods

We analyzed united atom MD simulation trajectories of unentangled, linear polyethylene (PE) melts with an increasing number of units (*N =* 10, 16, 30, 44, 66, and 96), using data from the literature [25,26,32], and from our own simulations [33,34,35,36], where the entanglement molecular weight *N_e_* = 130. MD simulations were performed in the canonical ensemble in a cubic box with periodic boundary conditions in the three dimensions. More details on the simulations are available in the referenced papers [25,26,33,34,35,36]. Here, we present the results for two systems: *N* = 30 at density $\rho =0.0317094\;\mathrm{sites}/{\dot{A}}^{3}$ and temperature *T* = 400 K, and *N* = 96 at density $\rho =0.0328194\;\mathrm{sites}/{\dot{A}}^{3}$ and temperature *T* = 448 K. Both follow intramolecular Gaussian chain statistics. The first represents a system far from the entanglement crossover and the second a system close to it. All the samples and the chain lengths we studied display similar behavior so that the results of this analysis are general and pertinent to polymer melts. Note that polymer melts with different monomeric architectures show anomalous dynamics consistent with the results presented here [37].

## 3. Results

**Figure 1:**
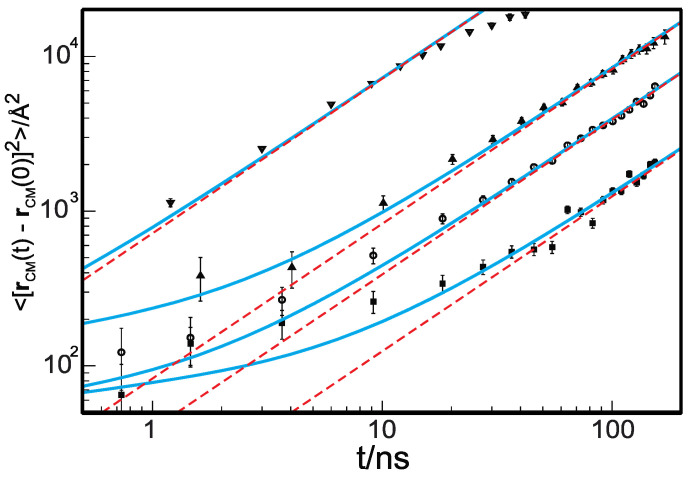
Mean-squared displacement of the center of mass of PE chains with an increasing degree of polymerization. Symbols are from neutron spin echo experiments for N = 36 (down triangles), 106 (up triangles), 192 (open circles), and 377 (filled squares). The light-blue lines are the predictions of the theory for cooperative dynamics (CD-GLE). The red dashed lines represent diffusive dynamics, i.e., the predictions of the Rouse theory. The vertical bars are the longest Rouse relaxation time. Data are reproduced with permission from reference [41] Copyright © 2008, American Chemical Society.

The single-chain Rouse equation correctly predicts the scaling with the degree of polymerization, N, of the diffusion coefficient, D, and shear viscosity [1,14]. However, the Rouse equation does not describe the anomalous dynamics observed in the short-time regime [14,23,38] when one samples the dynamics at time intervals $\Delta t<\tau_{Rouse}$, where $\tau_{Rouse}\sim R_{g}^{2}/D$ is the longest intramolecular relaxation time. Thus, the longest Rouse time is the time a molecule requires to diffuse a distance comparable to its dimension, $R_{g}$. The radius of gyration, $R_{g}$, is defined for long chains (obeying Gaussian statistics) as $R_{g}^{2}=Nl^{2}/6$, and *l* is the statistical segment length. The Rouse equation is compatible with a freely jointed chain representation [1], where $\langle \vec{l_{i}}\cdot \vec{l_{j}}\rangle =0$ for i≠j and i,j=1, …, N [39]. Experimentally observed anomalous dynamical behavior in unentangled polymer melts includes (i) subdiffusive center-of-mass (c.o.m.) dynamics, (ii) anomalous segmental diffusion, and (iii) stretched exponential decay of local and global normal modes of motion. Figure 1 displays the mean-squared displacement of the center of mass of polyethylene, measured at T=509 K, density $\rho =0.733\;\mathrm{gr}/cm^{3}$, and with an increasing degree of polymerization, $N=36\;\left(\tau_{Rouse}=0.85\;\mathrm{ns}\right),$ $N=106\;\left(\tau_{Rouse}=21.79\;\mathrm{ns}\right),$ $N=192\;\left(\tau_{Rouse}=83.69\;\mathrm{ns}\right)$, and $N=377\;\left(\tau_{Rouse}=534.08\;\mathrm{ns}\right)$, measured by neutron spin echo (NSE) [40,41]. The symbols represent the experimental data, and the red dashed lines are the Rouse diffusive c.o.m. dynamics. Clearly, NSE experiments show that the dynamics at times shorter than the Rouse longest relaxation time are subdiffusive.

**Figure 2:**
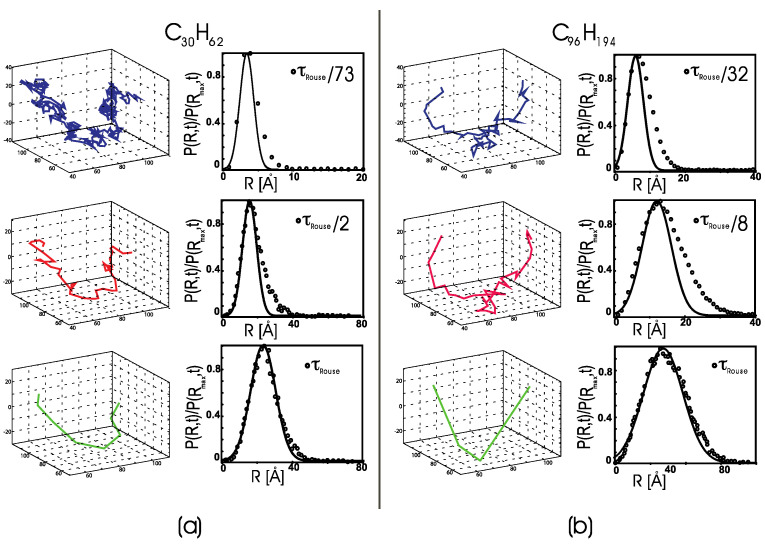
(**a**) Left panels: the MD trajectory of a single polymer’s center of mass in a melt of C_30_H_62_ polyethylene chains. The trajectory is sampled every $\Delta t=\tau_{Rouse}/73$, $\Delta t=\tau_{Rouse}/2$, and $\Delta t=\tau_{Rouse}$ (from top to bottom), where $\tau_{Rouse}\sim 730\;\mathrm{ps}$ for C_30_H_62_. Right panels: the normalized distribution of center-of-mass mean-squared displacements, sampled at the same time intervals sampled in the left panels. Lines: the best Gaussian fits of the slow part of the distributions. (**b**) The MD trajectory (left) and normalized distribution of center-of-mass mean-squared displacements (right) for a melt of C_96_H_194_ polyethylene chains, shown using the same convention as in Figure 1a. The trajectory is sampled every $\Delta t=\tau_{Rouse}/32$, $\Delta t=\tau_{Rouse}/8$, and $\Delta t=\tau_{Rouse}$ (from top to bottom), where $\tau_{Rouse}\sim 12\;\mathrm{ns}$ for C_96_H_194_. Lines: best Gaussian fits of the slow part of the distributions.

The direct observation of the center-of-mass trajectories from MD simulations, sampled for $\Delta t<\tau_{Rouse}$, shows that every single polymer’s c.o.m. undergoes transient periods of small-amplitude motion followed by periods of large-amplitude displacements (see an example in Figure 2). At any instant, molecules are partitioned between mobile and less mobile sub-populations, but the identity of the molecules assigned to either group changes in time. These features recall the anomalous dynamics observed in undercooled “fragile” glass-forming liquids. Thus, we apply the conventional analysis of anomalous dynamics for undercooled liquids to the trajectories from the polymer melt simulations. Note that the temperatures in the canonical simulations are higher than the glass transition temperature of linear polyethylene ($T_{g}\sim 148\;K$) [42].

To quantitatively characterize the heterogeneous dynamics as a function of the length of the time interval, we calculated the distribution of the center-of-mass displacement for each molecule *i*, $R_{i}\left(t\right)=||{\vec{r}}_{CM}^{i}\left(t_{2}\right)-{\vec{r}}_{CM}^{i}\left(t_{1}\right)||$*,* at a given time interval $t=t_{2}-t_{1}$, where, for convenience, we set $t_{1}=0$. The displacement distributions should follow a single-mode Gaussian function if the center-of-mass dynamics were purely diffusive (i.e., a Brownian motion). Figure 2a compares P(R,t) for a melt of C_30_H_62_ chains to a sample chain’s center-of-mass trajectory, where both are analyzed at the same fixed time interval.

We start from a time interval much shorter than $\tau_{Rouse}$ and then we increase the time interval until we reach the longest Rouse relaxation time (from top to bottom in the figure). 

When Δt is small and corresponding to a fraction of $\tau_{Rouse}$ (here, $\Delta t=\tau_{Rouse}/73∼10\;\mathrm{ps}$), the motion of the molecule’s c.o.m. alternates from fluctuations in a limited spatial region, i.e., the so-called “caged” dynamics, to large, directional displacements. For larger time intervals with $\Delta t<\tau_{Rouse}$, the trajectory is still dynamically heterogeneous, with alternating periods of confined and free dynamics. The corresponding distributions, *P*(*R*,*t*), show a non-Gaussian tail in the large-displacement region due to fast dynamical processes, which is the second signature of heterogeneous dynamics. At $\Delta t=\tau_{Rouse}$ ($\tau_{Rouse}\sim 730\;\mathrm{ps}$ for C_30_H_62_), the distribution becomes Gaussian, and the c.o.m. motion becomes diffusive. Accordingly, the dynamical heterogeneities are averaged out at this time interval, and the c.o.m. trajectory follows a Brownian motion.

Figure 2b displays a similar behavior for the C_96_H_194_ sample. The normalized probability distribution shows that some molecules undergo a slow diffusive motion, represented by a Gaussian distribution when sampled at a short time interval. In contrast, others undergo a fast large-displacement diffusion, corresponding to the tail in the weighted probability distribution. The nature of the slow diffusive motion becomes clear when one compares the distributions sampled at increasing time intervals. At $\Delta t=\tau_{Rouse}$ (bottom panels), the polymer c.o.m. trajectory becomes diffusive, and the distribution of displacements is well represented by a Gaussian function. Note that the anomalous dynamics trajectory follows a three-dimensional path, which appears to be different from a Lévy flight (not a heavy-tailed distribution). The trajectory suggests a complex free energy landscape with several minima, where the molecule tends to localize (small displacement), and which are separated by energy barriers that the molecule overcomes, thereby undergoing large displacements. This description agrees with the probability distribution, P(R,t), where one observes at least two populations, one with molecules undergoing slow dynamics and a tail with molecules undergoing fast dynamic. At the given time interval of sampling, the logarithm of the probability distribution, $F\left(R,t\right)=-k_{B}T\;\log P\left(R,t\right)$, gives the normalized free energy as a function of the chain displacement. Thus, the population of chains that undergo large displacements is smaller and energetically less favorable than the population undergoing a slow diffusion. Further analysis of the energetics is reported towards the end of this manuscript, where we show how rare, large-scale displacements correlate with energetically unfavorable chain stretching for the C_96_H_194_ sample.

Note that the trajectories reported here for one chain represent the dynamics of all the polymers in the simulation box and are similar for all the simulated systems. We observe that, while the polymers are partitioned into slow- and fast-diffusing molecules when sampled at short time intervals, for a time interval $\Delta t\geq \tau_{Rouse}$, the population becomes uniform, and the system becomes ergodic. The features detected in this analysis are in accord with the mechanisms of anomalous dynamics observed in undercooled “fragile” glass-forming liquids [43,44]. However, our fragile systems are not undercooled. The presence of these dynamical anomalies seems to be related to the competition between chain connectivity and intermolecular excluded volume interactions, which induces frustration and an anomalous slowing down of global dynamics even far from the glass transition. The competition between these two effects is unique to polymer melts and polymers in concentrated solutions, and is the physical origin of the “correlation hole” in the structure of polymer melts [45,46].

**Figure 3:**
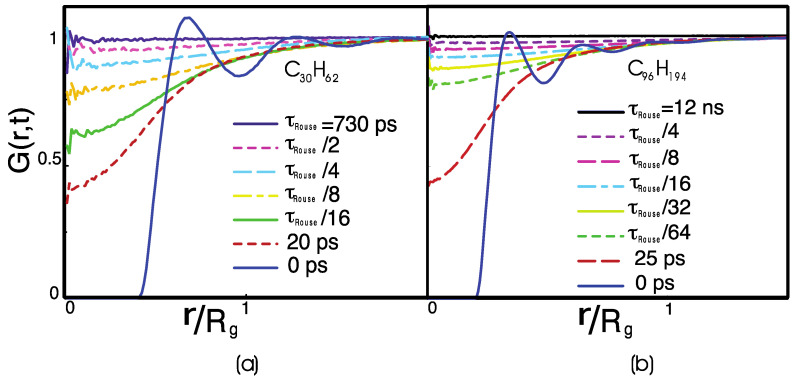
The monomer-distinct part of the van Hove function for a melt of (**a**) C_30_H_62_ and (**b**) C_96_H_194_ polyethylene chains. In (**a**), the van Hove function is calculated for $\Delta t=\tau_{Rouse},\;\frac{\tau_{Rouse}}{2},$ $\frac{\tau_{Rouse}}{4},\frac{\tau_{Rouse}}{8},\frac{\tau_{Rouse}}{16},\;20\;\mathrm{ps},\;0\;\mathrm{ps}$ (from top to bottom), where $\tau_{Rouse}\sim 730\;\mathrm{ps}$ for C_30_H_62_. In (**b**), the van Hove function is calculated for $\Delta t=\tau_{Rouse},\;\frac{\tau_{Rouse}}{4},$ $\frac{\tau_{Rouse}}{8},\frac{\tau_{Rouse}}{16},\frac{\tau_{Rouse}}{32},\;\frac{\tau_{Rouse}}{64},\;\;25\;\mathrm{ps},\;0\;\mathrm{ps}$ (from top to bottom), where $\tau_{Rouse}\sim 12\;\mathrm{ns}$ for C_96_H_194_.

Figure 3 displays the van Hove distribution function, which at time Δt=0 is the equilibrium radial distribution function, g(r) [10]. At equilibrium, the probability of finding two monomers belonging to different chains at a distance r becomes 100% successful only at a distance larger than the polymer radius of gyration. In the meantime, the probability of finding another polymer at a distance smaller than $R_{g}$ is finite, i.e., it is not zero. These observations indicate that polymers interpenetrate inside the “correlation hole,” which is the spherical volume defined by the polymer radius of gyration, $V\sim R_{g}^{3}=N^{3/2}l^{3}/6^{3/2}$. In that volume, there are statistically n=ρV/N interpenetrating chains, where ρ~1 in the monomer density. Thus, each polymer is in contact with $n-1\propto N^{1/2}$ other polymers at any time in the simulation.

$\tau_{Rouse}$ is the time needed for a chain to escape from its correlation hole, completely renewing its local contacts. It is also the characteristic relaxation time of the dynamical heterogeneities (Figure 1). Note that a time of the order of $\tau_{Rouse}$ is also needed to equilibrate a polymer simulation. The related length scale of $R_{g}$ corresponds to the average size of the dynamical heterogeneities (~0.8 nm for C_30_H_62_ and ~1.6 nm for C_96_H_194_). 

In Figure 3, the monomer van Hove distribution function shows two different characteristic times of relaxation. The first defines the time necessary for the fluid to lose the memory of its initial configuration on the local scale, corresponding to an intermolecular distance r~l, and to a short relaxation, ~20 ps, which is needed for the loss of the fine monomeric structure. This process is identical for C_30_H_62_ and for C_96_H_194_, and it is molecular weight-independent. In fact, on the local scale, the monomer dynamics depend on the chain stiffness and the local monomer density. Still, it is independent of the polymer length unless the chain’s length is short and comparable to the persistence length. The monomer relaxation, however, is sensitive to the specific chemical structure of the monomer, i.e., the local conformational energy barriers. Its timescale depends on the chemical structure of the monomer [15,47]. On the global scale of $R_{g}$, i.e., of the correlation hole, the van Hove function decays on a timescale comparable to $\tau_{Rouse}$. Thus, this second timescale is N dependent. The decay is bounded by an intermolecular distance of the order of the correlation hole, $r\sim R_{g}$.

The effective, time-dependent intermolecular mean force potential associated with the van Hove function is defined as $W\left(r,t\right)\propto -k_{B}T\;\ln G\left(r,t\right)$. This potential couples the single-chain dynamics with the motion of the chains surrounding the “tagged” chain inside the correlation hole. The motion of two molecules can be considered to be uncorrelated only when their dynamics are sampled at a distance larger than the range of the potential, $\Delta R>R_{g}$, i.e., the correlation hole, or on a time interval $\Delta t>\tau_{Rouse}$. Because $\tau_{Rouse}$ is also the longest intramolecular relaxation time, this simple analysis suggests that there is no separation of timescales between the relaxation of a given macromolecule and the relaxation of its surroundings. Thus, the leading hypothesis that justifies the derivation of the generalized Langevin equation, and the Rouse equation, from the Hamiltonian of the melt by Mori–Zwanzig projection operator is not fulfilled in the case of isotropic, uniform, one-component liquids, including polymer melts. 

Thus, the dynamics of a given chain is coupled through a time-dependent potential of mean force to the motion of its surrounding matrix in the timescale $\Delta t\leq \tau_{Rouse}$. This observation explains the inability of the mean-field Rouse equation to correctly predict the dynamics in the timescale shorter than the time necessary for the coupling potential to decay to zero. Therefore, the following natural step is the derivation from the Liouville equation of the dynamics of a group of interacting chains, where the motion is coupled by the time-dependent potential of mean force just observed. If one follows these steps and solves the Langevin equation for a group of $n^{\prime }$ interacting polymers, one can derive a set of coupled Rouse-like equations that predict anomalous dynamics [29,30,31]. In this model, which we called the cooperative dynamics generalized Langevin equation (CD-GLE), there is one free parameter, $n^{\prime }$, which is optimized by direct comparison with data of c.o.m. mean-squared displacement. When the theory is compared with data of mean-squared displacement from simulations or from experiments, one finds that the optimal number of correlated chains obeys the predicted scaling of $n^{\prime }\propto N^{1/2}$, which is in agreement with the simple analysis just presented. For example, for the data in Figure 1, one finds that for $N=36,\;n^{\prime }\sim 2$, $N=106,\;n^{\prime }\sim 4$, $N=192,\;n^{\prime }\sim 9$, and $N=377,\;n^{\prime }\sim 12$ [41].

Finally, we analyze the mechanism that leads to the interchanging between slow- and fast-diffusing center-of-mass motion. We see that the fast cooperative dynamics involve long-range unidirectional center-of-mass displacements that are correlated with the appearance of intramolecular polymer configurations having a high percentage of trans (stretched) conformations (~40% of bonds in the chain, as shown below). Thus, we calculate the square end-to-end distance for the average conformation assumed by each molecule i during a given time interval Δt. 

**Figure 4:**
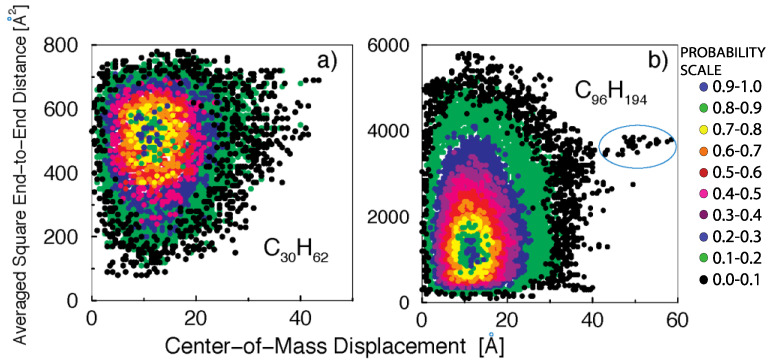
Contour plots of the normalized joint distribution for center-of-mass displacements and averaged square end-to-end distances: (**a**) for a melt of C_30_H_62_ polyethylene chains during a time window $\Delta t_{max}=365\;\mathrm{ps}$; (**b**) for a melt of C_96_H_194_ polyethylene chains during a time window $\Delta t_{max}=1500\;\mathrm{ps}$. The encircled region highlights the presence of persistent stretched configurations that facilitate the enhanced chain displacement.

In Figure 4, we plot the joint distribution of center-of-mass displacements and the averaged square end-to-end distances for the time interval that maximizes the contribution due to fast dynamical processes, as determined from Figure 2 ($\Delta t_{max}=\tau_{Rouse}$/2 for C_30_H_62_ and $\Delta t_{max}=\tau_{Rouse}$/8 for C_96_H_194_). The different colors represent the contours of the three-dimensional normalized distribution function. The melt of short polymer chains (Figure 4a) clearly shows that fast dynamical relaxation is correlated with the appearance of persistent all-trans configurations. This correlation cannot be explained by the Rouse model, where center-of-mass diffusion and internal dynamics (stretching) are rigorously uncoupled when the friction is uniform along the chain (non-uniform friction is known to couple rotation and translation dynamics with internal fluctuations). 

In the C_30_H_62_ sample (Figure 4a), the mean-squared end-to-end distance is $<R_{ete}^{2}>=480\;{\dot{A}}^{2}$, while a fully extended chain would have $<R_{ete}^{2}>{}_{extended}={\left(30\cdot 1.54\right)}^{2}\;{\dot{A}}^{2}=2134\;{\dot{A}}^{2}$. The persistent stretched configurations, $<R_{ete}^{2}>\sim 700\;{\dot{A}}^{2}$, correspond to ~45% of chain stretching. For the C_96_H_194_ (Figure 4b), where $<R_{ete}^{2}>=1536\;{\dot{A}}^{2}$, the stretched configurations with $<R_{ete}^{2}>\sim 4000\;{\dot{A}}^{2}$ correspond to ~36% of chain stretching.

Completely stretched configurations are entropically unfavorable and become less probable with increasing polymer lengths. In fact, for C_96_H_194_ the appearance of stretched configurations becomes a rare event, as exemplified by the isolated group of dots encircled in Figure 4b, which parts from the main distribution. This observation highlights the presence of microscopic mechanisms of dynamical relaxation in polymer melts that become increasingly suppressed with the increasing polymer molecular weight.

An analysis of the molecules undergoing fast diffusion shows that they follow cooperative mechanisms of motion, both inter- and intramolecular in character. Large-scale intramolecular displacements involve a string-like motion of monomers comprising the polymer chain, which corresponds to local chain stretching, as shown in Figure 4. Fast intermolecular cooperative dynamics can occur in one of two ways: (i) a single polymer undergoes a large displacement to a nearest-neighbor position, while a second polymer immediately replaces its initial position with the two chains following each other (a mechanism analogous to the one observed in undercooled colloidal fluids); or (ii) neighboring polymer chains may also diffuse by stretching and moving together, following parallel trajectories to final neighbor positions (a new mechanism pertaining to polymer fluids). Given that fast motion occurs via the partial stretching of the chains, which is entropically unfavorable, the pairing of two chains facilitates intermolecular packing, increasing the entropy of the surrounding chains by freeing the free volume in a mechanism that is reminiscent of a depletion effect.

At higher molecular weights, the effect of entanglement takes place, where the dynamics present scaling exponents different from the unentangled dynamics [1]. Still, in the motion occurring “inside the tube,” one can detect the same anomalous chain dynamics observed in unentangled chains with a subdiffusive c.o.m. motion. The application of a theory for many chains interacting through a time-dependent potential of mean force where entanglements are present shows a remarkable agreement with the relaxation of the dynamic structure factor, as measured by experiments of the neutron spin echo [48,49,50].

## 4. Conclusions

The microscopic analysis of simulation trajectories presented here supports the concept that the observed anomalous dynamics of a single chain in a polymer melt is related to the presence of interacting and interpenetrating chains inside the chain’s correlation hole. The distinct part of the van Hove function shows that intermolecular contributions are relevant in the global single-chain dynamics in polymer melts because of the mean-force potential. This observation suggests that single-chain models, such as the Rouse model, may be limited when describing chain dynamics in the range of timescales and length scales where the intermolecular potential of mean force is active ($\Delta t\leq \tau_{Rouse}$). In fact, the range and timescale of the time-dependent intermolecular potential of mean force define a region in space and time where the onset of dynamical heterogeneities is detected. We also observe novel mechanisms of fast intermolecular cooperative dynamics that involve chain stretching and, thus, are specific to polymer melts. These mechanisms are coupled to entropy-sensitive, conformational transitions from coiled to stretched polymer configurations. 

The observed separation of timescales between slow- and fast-relaxing domains in the short time regime justifies the projection of the dynamics of the fluid onto the coordinates of a group of slow, interacting molecules, obtaining, in this way, a set of coupled equations of motion for the region of slowly rearranging dynamics that reproduce well the observed anomalous dynamics [49,50]. The analysis presented here formally connects the observed anomalous dynamics to microscopic mechanisms of heterogeneous dynamics and provides a consistent picture of the physical phenomena underlying the observed anomalous, slow center-of-mass diffusion in polymer melts.

## Data Availability

Simulation trajectories from references [25,26,32] were kindly shared by the authors. Our simulation trajectories for PE at increasing degree of polymerization are available upon request.
